# Genetic Diversity, Population Structure, and Parentage Analysis of Croatian Grapevine Germplasm

**DOI:** 10.3390/genes11070737

**Published:** 2020-07-02

**Authors:** Maja Žulj Mihaljević, Edi Maletić, Darko Preiner, Goran Zdunić, Marijan Bubola, Eva Zyprian, Ivan Pejić

**Affiliations:** 1 Department of Plant Breeding, Genetics and Biometrics, Faculty of Agriculture, University of Zagreb, Svetošimunska 25, 10000 Zagreb, Croatia; ipejic@agr.hr; 2 Department of Viticulture and Enology, Faculty of Agriculture, University of Zagreb, Svetošimunska 25, 10000 Zagreb, Croatia; emaletic@agr.hr (E.M.); dpreiner@agr.hr (D.P.); 3 Centre of Excellence for Biodiversity and Molecular Plant Breeding (CoE CroP-BioDiv), Faculty of Agriculture, University of Zagreb, Svetošimunska 25, 10000 Zagreb, Croatia; 4 Institute for Adriatic Crops and Karst Reclamation, Put Duilova 11, 21000 Split, Croatia; goran.zdunic@krs.hr; 5 Institute of Agriculture and Tourism, Karla Huguesa 8, 52440 Poreč, Croatia; marijan@iptpo.hr; 6 JKI Institute for Grapevine Breeding Geilweilerhof, 76833 Siebeldingen, Germany; eva.zyprian@julius-kuehn.de

**Keywords:** grapevine, Croatia, genetic diversity, genetic structure, parentage, synonyms, SSR, SNP

## Abstract

Croatian viticulture was most extensive at the beginning of the 20th century, when about 400 varieties were in use. Autochthonous varieties are the result of spontaneous hybridization from the pre-phylloxera era and are still cultivated today on about 35 % of vineyard area, while some exist only in repositories. We present what is the most comprehensive genetic analysis of all major Croatian national repositories, with a large number of microsatellite, or simple sequence repeat (SSR) markers, and it is also the first study to apply single nucleotide polymorphism (SNP) markers. After 212 accessions were fingerprinted, 95 were classified as unique to Croatian germplasm. Genetic diversity of Croatian germplasm is rather high considering its size. SNP markers proved useful for fingerprinting but less informative and practical than SSRs. Analysis of the genetic structure showed that Croatian germplasm is predominantly part of the Balkan grape gene pool. A high number of admixed varieties and synonyms is a consequence of complex pedigrees and migrations. Parentage analysis confirmed 24 full parentages, as well as 113 half-kinships. Unexpectedly, several key genitors could not be detected within the present Croatian germplasm. The low number of reconstructed parentages (19%) points to severe genetic erosion and stresses the importance of germplasm repositories.

## 1. Introduction

Domesticated grapevine (*Vitis vinifera* L. subsp. *sativa*) is one of the major fruit crops in temperate regions [1]. Most of the world’s vineyards are planted with varieties that have been perpetuated for centuries by vegetative propagation [2]. Until the beginning of the 20th century, the vast majority of these cultivars arose from seedlings of mainly unknown origin, and most of today’s well-known cultivars are their clonal offspring preserved throughout history [3]. Preference for a limited number of elite cultivars recognized worldwide for wine production and globalization of wine and table grape production, as well as market forces, have led to uniformity and loss of grape biodiversity. The structure of germplasm has been changing drastically; in the year 2000, half of the world’s vineyards were planted to 21 varieties, while, in 2010, this number decreased to only 15 varieties [4]. 

Estimation of genetic diversity and determination of the genetic relationships among the germplasm accessions, including parentage analysis, are the first steps in dissection of a crop’s genetics. Among the molecular markers used for this purpose, microsatellites, or simple sequence repeats (SSRs), have been the most utilized due to their well-documented advantages and are even today the most common system used for discrimination of grape germplasm, and even between individuals in human forensic investigations [5]. Recommendation of the common SSR set for cultivar identification [6,7], as well as integration of the results via a public European *Vitis* database [8], were key steps in the integration of the results obtained by various research groups [7,9,10,11]. 

The world’s leading grapevine collections have been extensively characterized during the past ~15 years, resolving issues of duplicates, homonyms, synonyms, establishing genetic relationships, and analyzing genetic structure of collected varieties. The largest collection is the National Institute of Agricultural Research (INRA) Vassal Collection in France [12,13,14,15,16]; the second largest is the Spanish Encin, IMIDRA (Instituto Madrileño de Investigación y Desarrollo Rural, Agrario y Alimentario) collection [17,18], followed by the Italian Cra-Vit in Conegliano [19], the German JKI collection at Geilweilerhof [7], the Italian FEM (Fondazione Edmund Mach) collection [20], and the USDA (United States Department of Agriculture) collection [21,22]. Other smaller winegrowing countries or regions have also genotyped their germplasm, primarily for identification of autochthonous varieties, like Austria [23], Portugal [24,25], Greece [26], Hungary [27], Transcaucasia [28], Slovenia [29,30], Bulgaria [31], Serbia [32], Bosnia, and Herzegovina [33], among others. The same has been done through international cooperation, e.g., for seven European countries [34], countries of the Iberian Peninsula [35], Southeast European countries [36], Western Balkan countries [37], and Eastern Europe [9].

The first alternative to SSR markers for fingerprinting was a set of 48 single nucleotide polymorphism (SNP) markers [38]. Although this set has been used by other researchers through direct comparison with IMIDRAs database [39,40], the SNP profiles obtained are not publicly accessible, even though they have been proposed for routine fingerprinting. Large grapevine collections have also genotyped their germplasm with SNP markers [20,41,42], and the most comprehensive SNP analysis of grapevine germplasm [43] is result of a large GrapeReSeq consortium that used the Vitis18KSNP chip and, consequently, suggested the use of 14 most informative SNPs for cultivar distinction.

Assignment of a variety to a certain gene pool helps to define its origin. In order to analyze it, it is necessary to include as much grapevine genetic diversity as possible, with as many informative markers as possible. This has primarily been achieved by genotyping of a larger number of representative cultivars [34,44] or large world collections [15,19,20,22,41,45]. The latest analysis [43] confirmed previously reported main divisions based on geography (Eastern-Western gradient) and human utilization (table vs. wine grape) and refined the structure through eight clusters.

It is very interesting to understand the events from the genetic point of view that resulted in today’s assortment. Grapevine varieties are part of each country’s cultural heritage and are often economically important. This knowledge is also essential for genome-wide association studies. Scientists have been interested in grape parentage reconstructions and kinship analysis since the late 1990s [46], and, in the last 20 years, full parentage validation or at least half kinship has been suggested in the analysis of large collections [14,19,41,43], also with the help of SNPs. In addition, parentages have been reconstructed in smaller national collections [10,47,48,49,50,51,52]. The use of chloroplast DNA microsatellites (chloroplast (cp)SSRs) facilitates the investigation of domestication and phylogeny of grape species [53], as well as in defining maternal genotypes in parentage reconstruction. 

Viticulture on Croatian territory dates back to Illyrian times [54], prior to the ancient Greek colonization that occurred in 4th century B.C. At the time when Croatian viticulture was most extensive (the late 19th and early 20th century), there were around 200,000 hectares and about 400 varieties in use, with more than 200 varieties in Dalmatia only [55]. Today, more than half of Croatian autochthonous varieties, listed a century ago, have been irreversibly lost due to various (historical) reasons. Conservation of grapevine genetic resources in Croatia started about 25 years ago and six germplasm collections were established [56]. Biodiversity of Croatian grapevine germplasm has been investigated by SSRs, to a certain extent, in terms of genetic identification and variability and clarification of synonyms and homonyms [36,57,58,59,60,61], genetic similarity to other germplasm [62], partial reconstruction of chlorotypes [53] and parentage reconstruction [63,64,65], and also partially indirectly as part of worlds’ largest collections [14,15].

Croatia never had its own domestic grape breeding program, nor is there any documentation of individual intentional crosses made. Croatian autochthonous varieties, which are still in cultivation or exist only in repositories, originate from the pre-phylloxera era. The assumption has been, therefore, that these varieties are mostly the result of spontaneous hybridization between pre-existing and/or introduced varieties in the area of present-day Croatia. 

The aim of this study was: (i) to fingerprint all available accessions, clarify synonymy, homonymy, and misnaming within Croatian grape repositories; (ii) to analyze genetic diversity and structure of Croatian cultivars; (iii) to determine and analyze chlorotypes distribution; (iv) and to infer the unknown and confirm the already known pedigrees. 

## 2. Materials and Methods 

### 2.1. Plant Material

**Figure 1:**
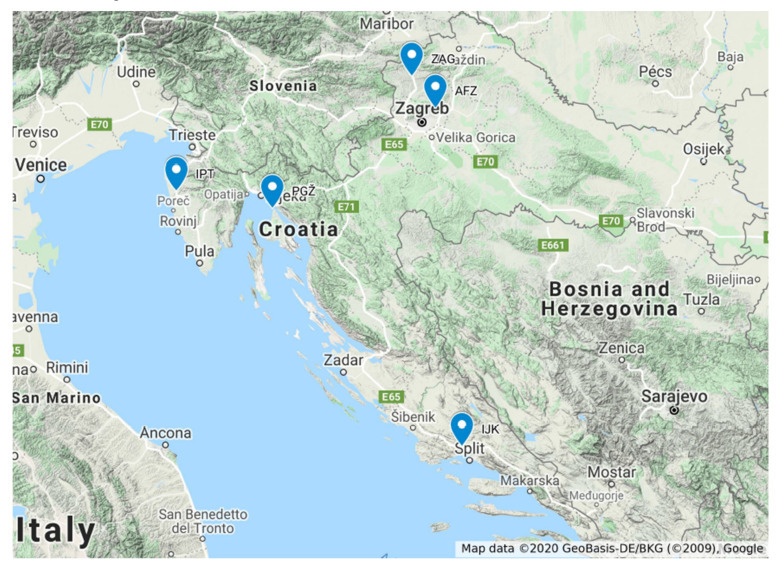
Geographical location and basic information on Croatian grapevine collections: AFZ—National Collection of Autochthonous Varieties, University of Zagreb, Faculty of Agriculture; Experimental station Jazbina, Zagreb; GPS: 45.857860, 16.003847; IJK—Collection of Dalmatian varieties, Institute for Adriatic Crops and Karst Reclamation; Split; Experimental station Duilovo, Split; GPS: 43.505778, 16.497970; IPT—Collection of Istrian varieties, Institute of Agriculture and Tourism Poreč; Poreč; GPS: 45.222686, 13.603673; PGŽ—Collection of Primorje-Gorski Kotar County; Risika; GPS: 45.097715, 14.640855; ZAG—Hrvatsko Zagorje grapevine collection; Donja Pačetina, Škaričevo; GPS: 46.111417, 15.869363.

The research included a total of 212 grapevine accessions, collected from all five national and regional grapevine collections, which are managed and maintained by public scientific institutions, with the exception of 7 accessions collected in situ, thus representing all Croatian grape germplasm. Geographical location of the collections within Croatia and their basic facts are presented in Figure 1. A list of grapevine accessions, including their codes, presumed varietal names, skin color, grape utilization, sampling collection, main cultivation region, conservation status, and types of analyses conducted, is given in Table S1. 

An additional set of 47 genotypes (*Vitis* SSR allele reference set), obtained from the Vassal collection, National Institute of Agricultural Research (INRA), France, was genotyped in order to ensure proper allele sizing and adjustment with external data sources (Table S2). The genotype ′Bombino bianco′, a variety which in previous studies [14] was shown to be related to the Croatian gene pool, was obtained from The Research and Innovation Centre, Fondazione Edmund Mach, S. Michele all’Adige, Italy. DNA extraction was performed using the peqGOLD Plant DNA Mini Kit (PEQLAB Biotechnologie GmbH, Erlangen, Germany) according to the manufacturer’s instructions.

### 2.2. SSR Genotyping

The selection of SSR loci was based on the level of their polymorphism, genome coverage, good amplification assessed through preliminary analyses, and compatibility with the previously published and available genetic profiles [66,67,68,69,70,71,72,73,74,75]. All selected loci were divided into four categories (cat.) according to their purpose (Table S3). Primers from cat.1 (which included nine SSRs proposed for cultivar identification [7]) were used for initial fingerprinting of all accessions and reference genotypes, cat.1 and cat.2 combined were used for analyses of genetic diversity and genetic structure, as well as parentage assignment, and primer pairs from cat.3 for analysis of genetic structure (just Croatian germplasm) and as additional support of reconstructed parentages. Cat.4 included primers needed for chlorotype determination. All SSR loci, except those from cat.1, were amplified using M13 (-21) tailed universal primers [76] in singleplex PCR for which the respective temperature regimes and PCR protocols were optimized. Additional data like primer sequences, associated annealing temperatures, and PCR and capillary electrophoresis arrangement of given loci are also presented in Table S3. 

PCR amplifications were carried out in a Veriti^TM^ Thermal Cycler (Applied Biosystems, Foster City, CA, USA). Multiplex PCR reactions were performed in a total volume of 10 μL containing 10 ng of DNA, 0.75 U Taq DNA polymerase (Sigma-Aldrich, St. Louis, MO, USA), 0.2 mM single dNTP, 0.3 μM forward and reverse primers, 2X PCR buffer (Sigma-Aldrich), 4.0 mM MgCl_2_, and 1 M Betaine solution, which served as a PCR enhancer. PCR temperature conditions were as follows: an initial denaturation of 2 min at 94 °C, followed by 35 successive cycles of denaturation (45 s at 94 °C), annealing (45 s at different T_a_ ranging from 50 to 60 °C, depending upon primer used), and elongation (60 s at 72 °C), as well as a final elongation at 72 °C for 20 min. After fingerprinting, a non-redundant set was defined and further genotyped at additional SSR and cpSSR loci. A unique M13 PCR protocol was used. Singleplex PCR reactions were performed in a total volume of 9.5 μL containing 10 ng of DNA, 0.75 U Taq DNA polymerase (Sigma-Aldrich), 0.21 mM single dNTP, 0.26 μM universal M13 and reverse primers, 0.068 μM forward primer, 1X PCR buffer (Sigma-Aldrich), 4.0 mM MgCl_2_, and 1.05 M Betaine solution. After an initial denaturation of 3 min at 94 °C, 29 consecutive cycles of denaturation (30 s at 94 °C), followed by 45 s of annealing and 1 min of elongation at 72 °C, were performed and then followed by 10 cycles of 30 s denaturation at 94 ° C, 45 s of annealing at 53 °C, and 1 min of elongation at 72 °C, with final elongation at 72 °C for 10 min. Amplified products were separated using an ABI3130 Genetic Analyzer (Applied Biosystems) with GeneScan- 500 LIZ^TM^ size standard. Sizing of the fragments was performed using GeneMapper 4.0 software (Applied Biosystems).

### 2.3. SNP Genotyping

SNP genotyping was performed on a set of non-redundant genotypes that differed to a lesser extent from that used in the SSR analysis due to technical reasons (highlighted in Table S1). The SNP set consisted of 47 SNP loci previously used and proposed for grapevine fingerprinting [38], to which the SNP locus (i.e., cp4527) for chlorotype determination was added [77] to validate the reactions (instead of locus SNP555_132). SNP loci names and allelic variants expected are listed in the (supplement data Table S4). A set of 47 SNP markers was run on a Biomark system applying the FluiDigm methodology. Primer pairs were used to amplify 47 DNA templates per run and one non-template control. The primers and fluorescently labeled oligo probes were designed and synthesized by FluiDigm (South San Francisco, CA, USA). Allele-specific PCR products were generated after pre-amplification of the target regions by duplex quantitative real time PCR using FluiDigm’s FR48.48 dynamic arrays. Genotypes were assigned based on the relative fluorescence intensities of the two alternative dyes (HEX and FAM) labeling the allele-specific probes targeting each SNP locus. Data analysis was performed in SNP Genotyping Analysis v4, and at least one negative control was included in the analysis for data normalization and auto-calibration (’no-template’ control - NTC normalization method). Obtained SNP profiles were compared with available published SNP profiles [40].

### 2.4. Microsatellite Database – Formation and Harmonization

Microsatellite profiles at nine common SSR loci were downloaded from the European *Vitis* database [8] for all available *Vitis vinifera* L. subsp. *sativa* accessions and adjusted using reference varieties and an Excel Macro Tool that converts actual allele lengths (bp) into encoded alleles, and vice versa, according to the principle previously described [6] and is freely available [78]. This resulted in the creation of a database of 2152 non-redundant genotypes, including 100 Croatian accessions previously genotyped through the GrapeGen06 project [7]. In addition, 1906 genetic profiles from publications containing shared loci [11,14,26,29,30,31,32,33,36,37,60,79,80,81,82,83,84,85,86,87,88,89,90,91,92,93,94] have been manually harmonized using common genotypes and added to the database for further analysis. Comparing all alleles across loci using the Excel-Microsatellite Toolkit [95], two conditions were identified: genotypes’ uniqueness and/or synonymy/duplication. Accessions were considered as duplicates when they had an identical SSR profile, and assuming the existence of genotyping errors/spontaneous mutations, maximum difference of two alleles was allowed. Redundant accessions were later considered and discussed as known/unknown synonyms but excluded from further analyses. A non-redundant genotype set that included all genotypes of putative Croatian origin was used for allele and chlorotype frequency calculations, analysis of genetic structure, and parentage reconstructions. Apart from the determination of synonyms, published genetic profiles [14] were used to back up analysis of genetic structure and parentage reconstruction. As M13 primers extend the amplicon length, standardization of the data was performed matching the length of alleles recorded in Reference [14]. 

### 2.5. Data Analysis 

Non-redundant data was analyzed for Polymorphism Information Content [96], null allele probabilities [97], and probability of identity (P(_ID_)), under the two assumptions: that the individuals within the test set are non-related (P_(ID)unrelated_), and, in the other case (P_(ID)sib_), that the individuals are related [98] as well as deviation tests from Hardy–Weinberg equilibrium (HW), were all calculated in Cervus [99]. In GenAlEx 6.503 [100,101], the average number of alleles per locus (N_a_) was calculated, as well as the number of effective alleles per locus (N_e_), expected heterozygosity, and observed heterozygosity (H_O_), and fixation index (F). Allelic richness (N_ar_) was calculated in FSTAT [102] using the formula of Reference [103]. The allele frequency calculation for SSRs, as well as SNP, markers (expressed as minor allele frequency) was performed in Microsatellite Toolkit [95]. Each Croatian accession was assigned to one of four regions, according to their cultivation area: Istria and the Croatian Littoral (*n* = 23), northwestern Croatia (*n* = 28), Slavonia and the Danube region (*n* = 1), and Dalmatia (*n* = 75), all detailed in Table S1. The genetic dissimilarity coefficients between each pair of genotypes were calculated as –ln(PSA), where PSA is the proportion of shared alleles distance [104] in the Microsat program [105]. The dissimilarity matrix was further used for clustering of genotypes with UPGMA (Unweighted Pair Group Method with Arithmetic Mean) algorithm (for SSR data). UPGMA construction and dendrogram visualization was done in MEGA software [106].

### 2.6. Genetic Structure

The model-based Bayesian method in STRUCTURE version 2.3.4 [107] was also used to imply the genetic structure and define the number of groups within the analyzed data set in order to assign Croatian germplasm to a certain ancestral group. The analysis was performed on a set of 771 accessions, which also included 664 individuals (defined as traditional) from Reference [14], together with Croatian assumed set genotyped at 20 SSR loci. Those that have been defined as modern are the result of deliberate crosses by man and as such interfere with the assessment of the natural structure of the population. Refined Vassal genotypes were categorized into countries/regions of their presumed origin (based on the ampelographic literature), respectively, as done in Reference [15]: Balkans—BALK; Eastern Mediterranean and Caucasus—EMCA; Iberian Peninsula—IBER; Apennine Peninsula—ITAP; Maghreb—MAGH; Middle and Far East—MFEAS; Croatia; Russia and Ukraine—RUUK, Western and Central Europe—WCEUR, and a group of undetermined varieties—“ND”. A total of 10 repetitions were performed for each assumed k that ranged from 1 to 10. Each repetition consisted of a burn in a period of 150,000 steps followed by 150,000 Monte Carlo Markov Chain iterations under the assumption of the admixture model and the correlated allele frequencies. The choice of the most probable number of groups (k) was carried out in the Structure Harvester [108] program by comparing the average probability estimators, calculated as ln [Pr (X | K)] for each k value, as well as by the ΔK calculation based on the change in the probability log of data between successive values [109]. CLUMPP v.1.1.2 [110] was used to align data between independent repetitions for each k using the “greedy” algorithm with 10,000 repetitions. Structure Plot [111] was used for the graphical representation of the groups. Individuals with Q ⩾ 75% of their genome were assigned to the group of question, while individuals with Q values < 75% were considered to be of admixed origin. In order to confirm the genetic structure estimated by Structure analysis, only individuals with Q values > 0.75, were included in the molecular variance analysis (AMOVA). AMOVA was performed in GenAlEx 6.503 on a genetic distance matrix also based on the proportion of shared alleles calculated in Microsat [105].

### 2.7. Parentage Analysis 

Parentage analysis was performed in Cervus [99] using a non-redundant set of genetic profiles comprised of the Croatian set (*n* = 127) and Vassal genotypes (*n* = 1095). Based on the results of allele frequency analysis, parent pair (gender unknown) simulation and analysis were performed and, subsequently, determination of “single parent – paternity” was done in Cervus in the same way, thus revealing first-degree relationship (with possible types of relationship—parent, offspring, full-sib, or half-sib). A million of offspring were simulated with a proportion of 0.01 sampled parents with the possibility of self-fertilization and the existence of relatives among potential parents. The critical LOD (logarithm of odds) value, with a higher confidence level (95%), obtained by simulation, was used as the criterion for parentage assignment, as well as allowing for no more than 5% mismatching (one out of 20 SSRs). In the case of successful parentage reconstruction, the maternal type was designated, when possible, using chlorotype data.

## 3. Results

### 3.1. Genetic Characterization of Croatian Grapevine Germplasm Repositories

The status of each studied accession was determined based on the genetic profile of 9 common SSRs compared versus the internal database and classified as follows: (I) unique genotype; (II) synonym/duplicate in Croatia; (III) synonym in neighboring countries; (IV) synonym in distant countries; (V) domesticated variety of known foreign origin; (VI) intruder—intruder found within the true to type vines; (VII) questionable varietal status—accession has a unique genotype, but, due to the lack of ampelographic or literature data, its varietal status is questionable; (VIII) mislabeled accession/incorrect collection—accession is planted under the wrong name in the collection and is not true to type (mostly, these accessions have never been fingerprinted); and IX) incorrect sampling of tissue for DNA analysis. Details for each accession can be found in Table S5. 

Among 212 accessions, 19 duplicates were identified between collections (or within a single collection), so only one genetic profile (with positive ampelographic identification) was included in the non-redundant set. The same was done for 26 cases of synonymy among analyzed accessions. All identified synonyms and duplicates are highlighted in Table 1. In addition, one case of homonymy identified as a variety of the Istrian collection called Plavina istarska (5IPT) shares the same name, but not the genetic profile, with the homonymous variety Plavina (4-C) grown in Dalmatia. After elimination of duplicates, synonyms, and intruders, the unique (non-redundant) set of SSR profiles consisted of 145 accessions. Within the collections, 145 different genotypes were detected, and, for 46 genotypes (32% of them), a synonym was found in one of the foreign database or scientific publications. Considering the status of each of the 145 unique accessions, all those that did not have synonyms, or do have synonyms abroad but their foreign origin is not strictly proven, were assumed as a “Croatian set” of varieties, totaling 127 of such accessions. Their SSR profiles for 36 loci are presented in Table S6.

### 3.2. Descriptive Molecular Statistics for SSR And SNP Data 

SRR alleles and their frequencies for non-redundant set are given in Table S7. The genotypic data were fitted into allelic classes based mostly on a difference of three or two base pairs, but there were some alleles with a consistent difference of just one base pair. In addition, novel alleles not previously reported were detected, e.g., a new second allele at locus VMC4f3 (246 bp) in ′Kraljevina′. However, no new alleles were found for any of the 9 common fingerprinting SSR loci. The genetic diversity parameters for a total of 36 analyzed SSR loci were calculated for the entire set of 127 assumed Croatian varieties (Table 2). 

Percentage of missing data was below 5% for most loci with exceptions of VViv37, Vchr4a, Vchr11b, and Vchr2b loci, which had somewhat lower average amplification rate. On average, nine alleles per locus were amplified, with values ranging from two (Vchr17a) to 15 for Vchr8b and VMC4f3, which also had the highest allelic richness (13.59). The effective number of alleles ranged from 1.47 for locus VVIn73 to 8.20 for locus VVMD28, with a mean of 3.9. The same loci were at the end of range for gene diversity (0.32 and 0.88) and observed heterozygosity (0.32 and 0.94). Mean H_O_ (0.71) and H_e_ (0.70) were almost the same. For Vchr8b and Vchr14b loci, the highest fixation index, deviation from the Hardy–Weinberg equilibrium, and the highest incidence of null alleles was estimated (so they were therefore excluded from further parentage analyses). All other alleles used in the parentage analysis had a satisfactorily low chance of null allele’s occurrence. In correlation with previous parameters, informativeness of the SSR loci expressed as polymorphic information content (PIC) ranged from 0.29 for VVIn73 to 0.78 for VVMD28, with the average PIC value of 0.66. Together with VVMD28, four more loci (ssrVrZAG79, ssrVrZAG62, VVMD5, and VVMD32) from the common set of nine SSRs were amongst the eight most informative ones. The single-locus P_(ID) unrelated_ values ranged from 0.027 (VVMD28) to 0.496 (VVIn73), whereas the P_(ID)sib_ values ranged from 0.318 (VVMD28) to 0.715 (VVIn73). The P_(ID) unrelated_ across all 36 loci was 1.19 × 10^−34^, whereas P_(ID)sib_ across loci was 5.29 × 10^−14^. 

SNP analysis was performed for the 124 genotypes (defined in Table S1) across 47 SNP loci (data provided in Table S8). Two loci were excluded from further analysis, one due to being non-polymorphic (SNP579_187) and the other one due to its poor amplification (SNP1445_218). Genetic diversity parameters were calculated for all remaining SNP loci (Table 3). The amplification success rate was very high, almost 100% except at the SNP259_199 locus, where only 0.81% of the data was missing. The lowest number of effective alleles (N_e_ = 1.03) was recorded for the SNP425_205 locus and the highest (2.0) for the five SNP loci (SNP197_82, SNP879_308, SNP945_88, SNP325_65, SNP1215_138), with mean of 1.71. The highest observed heterozygosity (H_O_ = 0.7) was detected for SNP581_114 and the lowest (H_O_=0.03) for SNP425_205. Significant deviation from the Hardy–Weinberg equilibrium was observed for SNP581_114 and VVI_10329, as well as a fixation index that was significantly greater or less than 0. At 9% of loci, the frequency of the minor allele was <0.1. The highest PIC (0.38) was observed for SNP879_308, SNP945_88, SNP197_82, and SNP325_65, while the lowest (0.03) was for the SNP425_205. The single-locus P_(ID) unrelated_ values ranged from 0.38 to 0.94 (SNP425_205), whereas the P_(ID)sib_ values ranged from 0.59 to 0.97, for the same loci, respectively. The P_(ID) unrelated_ across all 45 loci was 1.19 × 10^−34^, whereas P_(ID)sib_ across loci was 5.29 × 10^−14^. High frequency of null alleles (f > 0.05) was detected in 40% of the loci.

### 3.3. Comparison of SNP and SSR for Variety Discrimination

A set of nine SSRs, as well as set of 45 SNPs, both proposed for cultivar identification, were able to discriminate all investigated genotypes (data not shown). To compare the performance of nine SSRs and 45 SNP loci in variety distinction, a comparative summary of the genetic variation and polymorphism statistics (Table 4) is given. Amplification success was similar for both types of markers. The total number of alleles per locus (A = 92 for SSRs vs. A = 90 for SNPs) was almost identical. Expected and observed heterozygosity values were twice higher for 9 SSRs (e.g., H_O_ = 0.84 for SSRs vs. H_O_ = 0.416 for SNPs). Average PIC values were more than twice higher for SSR (0.76) compared to SNPs (0.31). However, cumulative P_(ID)sib_ values were more favorable in both cases for SNP markers (cum P_(ID)unrelated_ 4.57 × 10^−16^, cum P_(ID)sib_ 1.00 × 10^−08^) versus SSR markers (cum P_(ID) unrelated_ 4.07 × 10^−11^, cum P_(ID)sib_ 1.44 × 10^−04^). To obtain equal values of cumulative P_(ID)_ values for SNP markers, the 18–19 most informative SSR markers should be used. Having in mind these differences, nine common SSR markers were 2.5 times more informative than 45 proposed SNP markers. 

### 3.4. Chlorotype Distribution in Croatian Germplasm

Fragment lengths for the four analyzed cpSSRs and associated chlorotypes are listed in Table S9. Chloroplast DNA analysis on a set of 127 putative Croatian varieties resulted in the reconstruction of the chloroplast haplotype for 93% genotypes (Table S6).

**Figure 2:**
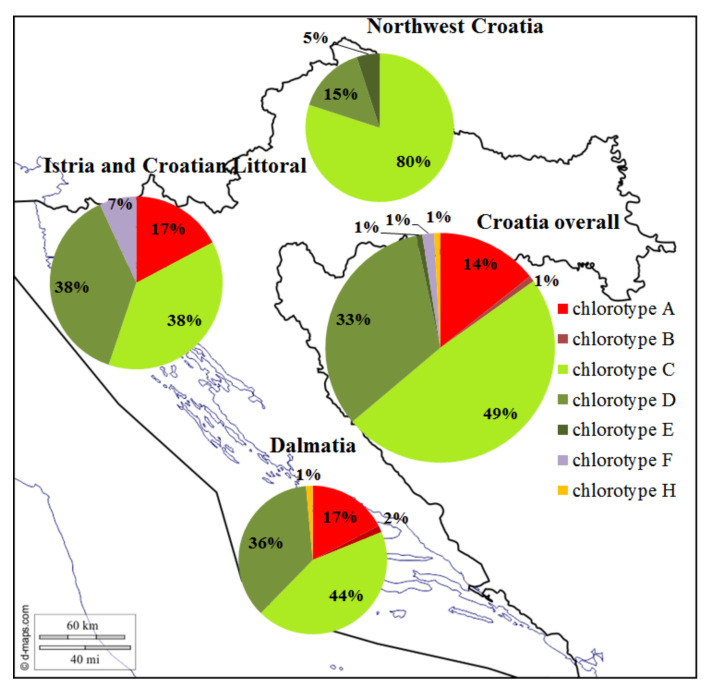
Graphical representation of chlorotype distribution across regions of Croatia.

A total of seven different chlorotypes (A, B, C, D, E, F, and H) were detected. Graphical representation of chlorotype shares across regions within Croatia (Figure 2) shows differences in their distribution. Generally, in Croatia, the most prevalent chlorotype is C, which occurs in about half of the varieties, one-third of varieties have chlorotype D, and the third most common has chlorotype A, with differences among regions in their geographical distribution. In northwestern Croatia, chlorotype C dominates and is followed by chlorotype D. Interestingly, chlorotype A, in which occurrence in coastal regions (comprising regions Istria and Croatian Littoral, as well as Dalmatia) accounts for 17%, was not recorded in northwestern Croatia at all. For the region of Istria and the Croatian Littoral, four different chlorotypes (A, C, D, and F) have been observed, with chlorotypes C and D being dominant. A total of five different chlorotypes (A, B, C, D, and H) have been recorded in Dalmatia, making it the region of highest chlorotype diversity. The dominant chlorotype in this region was chlorotype C. 

### 3.5. Cluster Analysis and Genetic Structure

**Figure 3:**
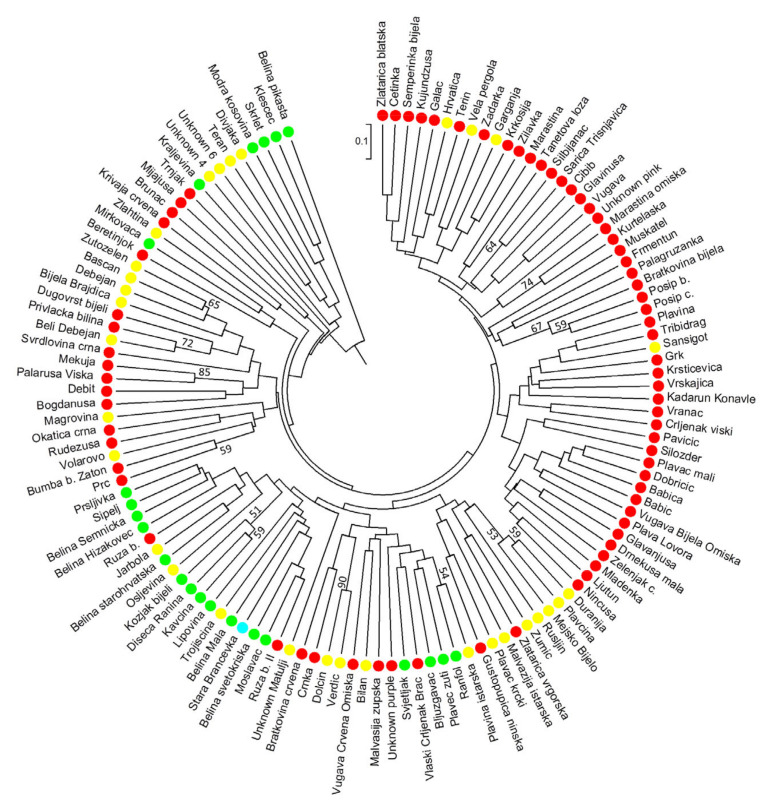
Circular UPGMA (Unweighted Pair Group Method with Arithmetic Mean) dendrogram for 127 presumably Croatian varieties based on 36 SSR loci. Dots represent varieties’ cultivation region: red = Dalmatia, yellow = Istria and the Croatian Littoral; green = northwestern Croatia; blue = Slavonia and Danube region.

The grouping of 127 presumably Croatian varieties based on genotyping with 36 SSR loci is shown in Figure 3. Clustering was mostly in correlation with regions of their cultivation. Dalmatian varieties are grouped separately, and, within existing subgroups, it is possible to notice grouping due to kinship, e.g., subgroups with ′Plavac mali′ or ′Tribidrag′. The same was true for the varieties of northwestern Croatia, for example, a subgroup around ′Belina starohrvatska′. Varieties from Istria and the Croatian Littoral also showed certain level of homogeneity within the sub-clusters, although their connections with both Dalmatian and continental varieties can be noticed.

Based on the Structure Harvester results, a decision was made on the best k (k = 5) as the number of original populations (Figure S1). Membership coefficients (Q values) in each of the five proposed populations are given in Table S10 for all putative Croatian varieties.

**Figure 4:**
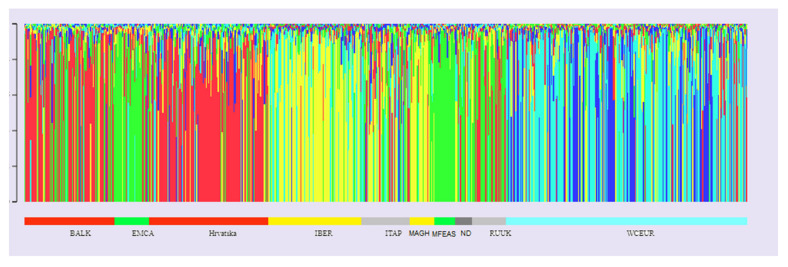
Graphical representation of the proposed structure for k = 5 according to Structure analysis. Each variety is represented by a vertical column colored according to the Q coefficient and the corresponding original population. The following abbreviations represent the sampling regions: BALK = Balkans; EMCA = Eastern Mediterranean and Caucasus; Hrvatska = Croatia; IBER = Iberian Peninsula; ITAP = Apennine Peninsula, MAGH = Maghreb region; MFEAS = Middle East and Far East; ND = a group of undetermined varieties; RUUK = Russia and Ukraine; WCEUR = Western and Central Europe. Balkan ancestral population is marked red, Iber ancestral population is marked yellow, East ancestral population is marked green, Biscay ancestral population is marked light blue, and Gallica ancestral population is marked dark blue.

The same is visualized in Figure 4, after the varieties have been sorted according to the sampling site or the assumed origin. With sampling location as the criterion, inferred original populations can be renamed as follows: population 1 (represented in red, marked Balkan) covers the largest number of varieties from Hungary, Croatia, Serbia, and Romania and could be broadly defined as the Balkans. Population 2 (represented in yellow and designated Iber) comprises the largest number of varieties from Spain, Portugal, Algeria, and Morocco and was defined as the Iberian Peninsula and Maghreb countries.

Population 3 (represented in green and labeled East) encompasses the countries of eastern Mediterranean and the Black Sea, as well as Middle and Middle East countries, such as Greece, Turkey, Bulgaria, Syria, Cyprus, and Uzbekistan. Population 4 (visualized in light blue color coded as Biscay) includes primarily varieties of southwestern France, i.e., varieties from Bordeaux, and a smaller number of varieties of northern Portugal, Galicia, and northern Spain, as well as those cultivated along the Bay of Biscay (offshore Atlantic). Population 5 (visualized in darker blue and designated Gallica) was formed primarily around two varieties, ′Pinot′ and ′Gouais blanc′, and their numerous offspring scattered throughout northeastern France and Germany. Out of the 127 putative Croatian varieties, 57 had Q ≥ 0.75 within population 1, designated Balkan. An additional 34 varieties had more than 50% of their genome originating in the Balkan set but are still considered to be admixed. A total of eight varieties had at least 75% of their genome originating in another population: ′Bogdanuša′, ′Maraština omiška′, and ′Frmentum′ originate from Iber population; ′Belina starohrvatska′ (syn. ′Gouais blanc′, ‘Heunisch weiss′) belongs to the Gallica group, while ′Cibib′, ′Krivaja crvena′, ′Mijajuša′, and ′Trnjak′ were assigned to the East group.

Varieties with Q values ≥ 0.75 (total of 455 varieties), distributed into five populations (Pop1 (Balkan) = 125, pop2 (Iber) = 87, pop3 (East) = 91, pop4 (Gallica) = 111, and pop5 (Biscay) = 41), were subjected to AMOVA. Results of AMOVA for the five subpopulations detected by Structure analysis (Table S11) confirm the proposed Structure clustering since Φ_PT_ was highly significant indicating the existence of a general structure and confirming that most genetic diversity results from differences between populations. Φ values for each population pair (Table S11) showed high significance for each pair of proposed populations and indicate that Balkan group is the most similar to East, while the most divergent is from the Gallica population.

### 3.6. Parentage Analysis

In Table 5, 24 proposed full parentages of Croatian varieties are presented, with 14 out of them having not been previously reported. The critical LOD values were determined by simulation of parentage (LOD = 14.5 for *P* = 0.05).

When possible, genotyping was further extended so number of matches (based on visual inspection) of additional 14 three- and tetra-SSR loci, as well as 45 SNP loci, are indicated. Chlorotypes, if known, are highlighted so that maternal genotypes are indicated where possible. Although cv. ‘Sokol′ was not part of 127 Croatian set, his already well-known parentage is shown, as confirmation of his foreign origin. All proposed parentages were significant for *P* = 0.05. Offspring detected resulted from cross pollination of two different varieties, and self-pollination was not detected. For eight parentages (′Belina starohrvatska′ included), both parents and offspring are part of Croatian germplasm, while, for the 17 proposed trios, at least one parent is of a foreign origin. The two most common varieties that emerge as a parent in six proposed trios are the Italian variety ′Bombino bianco′ (syn. Slovenian Trevolina [30]) and ′Belina starohrvatska′ (syn. French ′Gouais blanc′, German ‘Heunisch weiss′, Table 1). ′Bombino bianco′ can be characterized as a mother in at least half of the proposed trios. For two trios, ′Plavac mali′ is a second proposed parent, with ′Bombino bianco′ as a first, but since they share the same chlorotype, it is not possible to distinguish the maternal parent. The next most common parent in six crosses was ′Belina starohrvatska′, which was in half of the cases a pollen recipient from donor ′Bljuzgavac′ (syn. ′Blank blauer′ [14]), while its crossing with the Romanian ′Alba imputotato′ resulted in two more offspring. The aforementioned ′Bljuzgavac′ is part of four parentages, and parents—′Bratkovina crvena′ and Hungarian ′Gyöngy feher′—have been proposed for the same. The most represented Dalmatian variety was ′Plavac mali′ (for which previously proposed parentage [64] was not confirmed [112]), and it was recorded as one of the parents in four proposed trios. For three varieties (′Kozjak bijeli′, ′Muškatel′, and ′Volovina′), both proposed parents were of foreign origin.

A total of 113 half kinships (varieties sharing at least one allele at each of the 20 SSR loci) was reconstructed, among which: (i) 103 cases of putative direct (first-degree) relationships (with no possibility to determine if a cultivar is a parent, an offspring, or a full sibling of the second cultivar), out of which 61 relationships were not previously reported; and (ii) five half-sib relationships, of which two were not reported previously, as well as (iii) five not possible relationships due to the already known parentages, out of which two were not reported previously. The full list of first-degree relationships are given in Table S12, with some examples highlighted (Table 6). 

Varieties that are most interconnected within the presumable Croatian set through first-degree relationship are: ′Plavac mali′ with a total of twelve assigned relationships, ′Bombino bianco′ with a total of seven assigned relationships, ′Bljuzgavac′ with a total of seven and ′Tribidrag′ with a total of six assigned first-degree relationships. Within Croatian germplasm, for 71 varieties (56%) putative direct (first-degree) relationships was determined (most probably parent-offspring relationship) with one of the varieties from the total analyzed set. The aforementioned degree of relationship, but within Croatian germplasm only, was determined for 58 varieties (45%). For six varieties, ′Mijajuša′, ′Mladenka′, ′Muškatel′, ′Silbijanac′, ′Teran′, and ′Žutozelen′, possible first-degree relationships were determined exclusively with varieties of foreign origin.

## 4. Discussion

### 4.1. Determined Status of Croatian Grapevine Collections

Although Croatian germplasm has been genotyped via SSRs through various projects [36,57,58,59,60,61], this research represents the most comprehensive analysis of Croatian germplasm at the national level so far. Here, we covered all official grapevine repositories and defined latest status of all so far collected accessions in Croatia (Table S5). The process of germplasm collection requires years of reasoned management with occasional genetic verification of true to typeness, e.g., intruders have been identified within the National Collection of Autochthonous Varieties (Faculty of Agriculture in Zagreb) and were consequently excluded. Fingerprinting has proven particularly useful for the Collection of Istrian varieties (Poreč). Although collected in local vineyards through various conservation projects, Istrian accessions have not been adequately genotyped and compared with international databases [113]. Open issues of the mentioned collection were answered and defined mostly through mislabeled accession / incorrect collection (Table S5). Each germplasm collection will be able, based on the fingerprinting data, to review their duplicates/redundant or unnecessary accessions internally and make cost rationalization decisions. From the same data, the occurrence of duplicates between collections is visible, but such duplications are preferred for security reasons and, moreover, are standard procedure in most germplasm collections [114]. Finally, it should be noted that determining the status of "duplicates" in a collection using microsatellites does not necessarily mean that these accessions are phenotypically identical since they can represent different clones. Identified errors in the internal SSR database, as well as the revision and complementation of data on profiles at nine common SSR loci, enabled the correction of data for 20% of Croatian varieties in which profiles are stored within the largest public database of grapevine varieties, i.e., the European *Vitis* database. The accuracy and completeness of genetic profiles ensures proper estimation of the overall grapevines genetic variability [7].

### 4.2. Choice of Markers for Routine Fingerprinting 

Using the M13-tailed primers, VViv37 locus could not be amplified. Successful amplification of the VViv37 locus was achieved only with primers that were both fluorescently labeled. Since this problem was addressed at a later research stage, weak amplification of the aforementioned locus (Table 2) was probably due to DNA degradation caused over time. Optimization of the PCR reaction with the M13 approach (proposed as cost effective) showed to be challenging due to the method requirements, primarily its temperature regime [76] and length difference of the reverse and forward primer.

On average, the amplification rates for tri- and tetra-nucleotide loci were poorer compared to other loci, as well as their detected allelic richness, PIC, and P_(ID)_ values (Table 2), although the last three parameters were similar to those previously recorded [73]. In addition, the most polymorphic loci showed a high occurrence of null alleles, which has been observed also in other studies [10,73]. This can be an issue, especially in pedigree reconstructions where such loci need to be neglected. One of the main advocated advantages of three- and tetra-nucleotide markers is easier allele determination due to the smaller stuttering effect and easier binning. However, for certain loci, alleles different from the product of basic repeat number were detected, such as Vchr2b, Vchr11b, and Vchr16a locus (Table S7). As confirmed in the present study, irregularities in allele lengths at the Vchr11b and Vchr16a loci were identified earlier [73].

Loci recommended [13] and used in genotyping of world’s largest germplasm collection [14], in our study, proved to be equally effective, e.g., the two most informative loci in both studies were VVip31 and VVMD28. The cumulative P_(ID)sib_ value for the nine common SSRs today routinely used for genotyping was 1.44 × 10^−4^, and the same efficacy can be achieved using top eight most informative microsatellite markers (VVMD28, VVIp31, ssrVrZAG79, ssrVrZAG62, VViv67, VVMD5, VMC1b11, and VVMD32), except for the Vchr8b locus due to its high frequency of null alleles (Table 2). Given their high polymorphism, as well as other advantageous properties, like ease of amplification and well-known lab protocols and alleles expected, the recommended set of 9 SSRs proved once again to be a valuable and practical tool in grapevine fingerprinting.

### 4.3. Will SNP Markers Replace SSRs for Routine Identification of Grapevine Varieties?

In the last five years, SNPs have been the markers of choice in grapevine research, as well as for other crops [43,115,116,117]; however, still, the most used type of markers for routine fingerprinting are SSRs. An alternative to the common set of 9 SSRs in the form of highly reproducible and well scattered 48 SNP markers was proposed [36]. In this research, 47 of the 48 proposed SNP loci were analyzed, and 45 of them have been used for further analyzes. Although SNP579_187 locus was polymorphic in other studies [38,40] where minor allele frequency (MAF) value as 0.17 was reported [38], in this study, it was nonpolymorphic, i.e., uninformative for Croatian germplasm. This was less often the case with microsatellite loci since those selected for fingerprinting detected more than just two alleles per locus. PIC values were, on average, 2.5 times higher for 9 SSRs (PIC = 0.76) than for SNP markers (PIC = 0.31), which is in agreement with previous results [20,38,118]. Comparing the SNP and SSR loci based on P_(ID) unrelated_ (Table 4), it is evident that one SSR locus provides on average information as 3–4 SNP loci, respectively, which is identical to earlier findings [38]. However, bearing in mind the complexity of grapevine kinship [41], it is more appropriate to use a P_(ID)sib_ value indicating that 18 SSR markers provide the same information as 45 SNP loci, that is, one SSR locus provides information as 2.5 SNP loci. As genetic similarity among genotypes increases, so does the informative nature of the SNPs.

Large grapevine collections have genotyped their germplasm in the last decade [20,38,41], but resulting SNP profiles are generally not publicly available. It can be assumed that, without an international consortium initiative to form a public SNP profile database, international comparison will not be possible, as also stressed by others [43]. It is likely that smaller laboratories and research groups will continue to use microsatellite markers for routine genotyping (e.g., genotype checking in nursery production) due to lower equipment costs and the abundance of available microsatellite profiles of already analyzed grapevine varieties on a standard 9-loci set.

### 4.4. Links to Other Countries through Synonyms

For a third of genotypes within Croatian collections, a synonym was detected in one of the neighboring or more distant countries (Table 1 and Table S5). For a total of 95 accessions, no synonym has been detected in any other country, so we can consider them to be unique for Croatian germplasm (Table 1 and Table S5). Although the analysis of the genetic structure suggested that origin of four unique varieties (′Bogdanuša′, ′Trnjak′, ′Krivaja crvena′, and ′Cibib′) was from foreign gene pools, since they were not found in areas of their presumed origin and have a long history in Croatia, they can still be considered as part of the Croatian grapevine heritage. Conservation of these genotypes should be accentuated given their uniqueness. In addition, additional 7 varieties (′Garganja′, ′Draganela′, ′Hrvatica′, ′Dolčin′, ′Bilan′, ′Tribidrag′, and ′Bratkovina bijela′) might have Croatian origin because they have at least one Croatian variety as a first-degree relationship (most likely potential parent offspring pair), and other researchers have not reconstructed other first-degree relationships for the same. 

For varieties of coastal area, links with Italian, Slovenian, and, to a lesser extent, Greek germplasm have been established through identified synonyms. The similar results were suggested in earlier comparisons of germplasms [11,57,60,62]. Historically and climatically, these Mediterranean countries are interconnected by the Adriatic Sea. The influence of the Greek germplasm probably extends from the time of the first ancient Greek colonization of Adriatic islands [65] till today in presence of varieties like ′Mijajuša′ or ′Cipar′. Neighboring Bosnia and Herzegovina (B&H) is a country where many Croatian varieties are grown under the same name, e.g., as ′Plavac mali′ or ′Plavina′ from Croatia, or reciprocal example ′Žilavka′ from B&H in Croatia [33]. Synonyms of varieties from the continental part of Croatia have been confirmed primarily in Austria and Germany. The fact that numerous Muscat accessions grown in Croatia are synonyms of some well-known Muscat varieties has been confirmed in this study, as well as in previous ones, such as ′Muškat ruža porečki′ = ′Rosenmuskateller′ [57], ′Muškat momjanski′ = ′Muscat White′ [119], or ′Muškat ruža omiški′ = ′Muscat Hamburg′ [59,60]. To what extent Italian or Greek germplasm had impact on the formation of Croatian germplasm is evident from the established genetic structure and kinship, which will be discussed later. Numerous established and confirmed synonyms are probably the result of longtime migration of varieties via trade or migration routes. Migration direction is often difficult to prove, so their exact origin remains an open question. 

### 4.5. Chlorotype Variation and Geographical Distribution in Croatia 

The most extensive reconstruction of the chlorotypes of Croatian germplasm has detected seven different chlorotypes, revealed certain differences in their geographical distribution (Figure 2) and contributed to the affirmation of reconstructed pedigrees where chlorotypes of the two parents were distinct. Abundance in chlorotype diversity was found, including infrequent chlorotypes, such as E, F, or H, that were previously found specific for Near East *sylvestris* populations [53]. Chlorotypes of two accessions (‘Teran′ and ′Bogdanuša′) did not match those previously established [53] (Table S6). Determined chlorotypes allowed us to discriminate two varieties, ′Belina starohrvatska′ and ′Bombino bianco′, as key maternal genitors in the formation of Croatian germplasm. Although the three most extensive grapevine pedigree reconstructions [14,19,41] did not include chloroplast DNA analysis in their methodology, in this research, it proved to be a useful diagnostic and supportive tool in pedigree reconstructions. Greater diversity of chlorotypes in coastal Croatia is correlated with greater genetic variability determined at nuclear DNA level (data not shown). Continental Croatia is dominated by chlorotype C, the one which is also dominant for a group of French and German varieties [53]. Chlorotype A was not recorded for varieties of continental Croatia, although its share in coastal germplasm was 17%. Chlorotype A is a dominant genotype of European *sylvestris* populations, and its distribution in *sativa* populations diminishes from west to east [53]. One possible explanation for the absence of this chlorotype in the continental part is the dominance of ′Belina starohrvatska′ (chlorotype C) as the mother and the absence of *sylvestris* introgression. In contrast to our coastal part where chlorotype C is dominating, in Italy, chlorotype C is represented in a small percentage, with chlorotype D being the dominant one [53,120]. This is intriguing given the existence of numerous connections and links with Italian germplasm.

### 4.6. Genetic Diversity and Structure of Croatian Germplasm

Even though most grapevine varieties are self-fertilizing, they are highly heterozygous as also confirmed in this paper. Average observed heterozygosity calculated for Croatian germplasm (36 SSRs) was 0.71 (Table 2), but when considering only 20 SSR common loci used for same parameter calculated for the Vassal Collection (H_O_ = 0.76; *n* = 2096) [15], the H_O_ value was the same. Average number of alleles per locus (N_a_=9.6 for common 20 loci, data not shown; N_a_ = 9 for 36 SSRs) was expectedly lower than that calculated for entire INRA collection (N_a_ = 16.1). However, detected N_a_ = 8.9 for group S-3.3 (Wine = Balkans & East Europe, *n* = 226) [15] is somewhat lower than values of the Croatian set (N_a_ = 9.6). In fact, only eastern populations of table grape exhibit significantly more alleles per locus, so we can state that genetic diversity of Croatian germplasm is rather high considering its size. This is also supported by SNP genotyping results, where H_O_ values in this study (H_O_ = 0.416) were higher than those (H_O_ = 0.397) previously determined [38] for same loci (but for a diverse panel of varieties from Imidra (Spain) repository) and those (H_O_ = 0.349) determined by Reference [20] for a different SNP panel.

In order to gain insight into the genetic structure, it is necessary to include as many varieties as possible, as well as polymorphic, unlinked markers. Having in mind that the grape germplasm of a certain area does not represent necessarily a natural population, published profiles of only traditional varieties from the Vassal collection were included to reduce human impact through modern breeding. According to Evanno’s ΔK statistics (Figure S1) K = 5 was indicated as the most pertinent level of population subdivision and the assumed genetic structure was confirmed by molecular variance analysis (Table S11). As the optimal subdivision(s) of the Vassal collection, k = 3 and k = 5 were found [15]. For k = 5, two groups were defined as in the present study (Balkan and Iber group), and the largest difference was obtained for grouping of Western and Central European varieties that, unlike in this study (populations Biscay and Gallica), grouped into one group. In this study, 41% of varieties were admixed according to Q values. If we would use stricter criteria, Q≥ 0.85, as in Reference [15], the proportion of varieties of admixed origin for our established k rises to 53% versus 62%, in accordance with Reference [15]. The reason for the large percentage of varieties of admixed origin can be found in complex pedigrees and migration of grapevine material.

Almost half of the Croatian germplasm was assigned to population 1 designated Balkan (Table S10), in accordance with the eco-geographic grouping to proles Pontica (subproles *Balcanica*) proposed by Reference [121], thus confirming previous reports for a limited number of Croatian varieties that classified them as part of Balkan and East Europe [15] or Balkan only [43]. It is important to note that both continental and coastal varieties were grouped into the same Balkan population. Given the many links with Italian germplasm, it was expected that some varieties would be grouped with Italian ones. However, analyses of genetic structure [15,20] define the Italian population as admixed one and emphasize the role of the Roman Empire in the expansion of viticulture and, consequently, of varieties itself. The Balkan group shows the greatest similarity to the ancestral population East (Table S11), which agrees with previous results [43] and is the result of the grapevine migration via Mediterranean trade routes throughout history. 

Cluster analysis performed based on 36 SSRs (Figure 3) implies the existence of a structure within Croatian germplasm. A group of Dalmatian varieties is most homogeneous and formed around key varieties, such as ′Plavac mali′ and ′Tribidrag′, and the clusters follow established kinship relationships. In addition, a cluster containing both southern and northern Adriatic varieties grouped on the basis of kinship with the ′Bombino bianco′ variety is clearly distinguished, as well as a large cluster consolidating descendants of ′Belina starohrvatska′ and ′Bljuzgavac′. Varieties that were not part of Balkan ancestral group also exhibit lower genetic similarity with the rest of Croatian germplasm and are thus separated into different clusters, such as cluster of Eastern (presumably Greek) varieties: ′Mijajuša′, ′Krivaja crvena′, ′Trnjak′, and ′Brunac′. 

### 4.7. Origin of Presumably Croatian Varieties

All proposed parentages of Croatian varieties published in reference [14] were confirmed in this study, partly at additional SSR and SNP loci (Table 5). Full parentage was reconstructed for 24 varieties that make up 19% of the putative set of 127 Croatian varieties. For the Vassal collection, this was possible for as much as 35% of varieties [14]. The fact that almost twice as much pedigree trios could be detected for the worlds’ largest grapevine collection can be partly explained by the fact that half of them refer to modern varieties, created in the last 150 years, mostly through crossing of well-established and widespread varieties, which can, of course, still be found in germplasm collections. Discovery and confirmation of numerous parentages within French germplasm speaks in favor of well-preserved regional germplasm and suggests that inclusion of regional varieties within a candidate parent set increases the likelihood for successful parentage reconstruction. Published SSR profiles [14] originating from neighboring countries, such as Italy and Hungary, represent only a small fraction of their germplasm, whilst some countries, such as B&H or Serbia, are represented with just a few, and neighboring Slovenia with no accessions. In only five parentages, all three members were part of Croatian germplasm (not including ′Belina starohrvatska′), indicating that the number of reconstructed parentages could be higher if broader regional germplasm would be included. For a total of 45% of the varieties, at least one first-degree relationship (most probably parent-offspring relationship) with one of the supposed autochthonous varieties was detected, and for some of them first-degree relationships with foreign varieties were also detected. Therefore, assumption that most of the Croatian germplasm included in the research originated on the territory of present-day Croatia could not be confirmed, although most of the varieties today exist exclusively here. The low number of reconstructed parentages points to severe genetic erosion of Croatian germplasm since the late 19th century, as well as to the importance and justification for preserving neglected local varieties, although most of them are not economically interesting at present.

## 5. Conclusions

This study is so far the most representative and comprehensive analysis of Croatian grape germplasm. Although Croatia is a small wine growing country, the grape genetic diversity of its germplasm is high. SSR marker data enabled identification of many duplicates and synonyms both within and between collections, which will improve curating the germplasm repositories. The selected 45 SNP loci, previously recommended for fingerprinting, have proven to be a good identification tool, but their future relevance remains questionable due to the absence of public databases for comparison. So, we believe that the broadly accepted 9 SSRs set will remain the gold standard in varietal fingerprinting. The inferred genetic structure has classified 50% of Croatian germplasm as part of the Balkan group of cultivars in accordance with the eco-geographic grouping. The large number of admixed varieties is a consequence of complex pedigrees and migration of varieties over wider territory surrounding Croatia today. For a total of 95 accessions, no synonym has been detected in any other country, so we can consider them to be unique to Croatian germplasm. However, complete parentage reconstruction was possible for only 19% of varieties. This confirms the severity of genetic erosion in Croatian germplasm since the end of the 19th century. Contrary to the expectations, foreign varieties, i.e., genotypes that we failed to find within present day Croatian germplasm, participated in many parentages, and the number of cases where both parents are exclusively of Croatian origin is rather low. Thus, considering the rich political history of the entire region and changes of administrative borders, vicinity and similarity with particular geographical and agro-ecological areas of neighboring countries seems to be more relevant for genetic structure of germplasm than administrative borders of single countries. Studied varieties had been derived from spontaneous crosses of the same parental combinations or just with few key genitors, rather than out of many chance combinations. The empirical selection process that has been performed in the past by humans was probably a result of the unconscious preference for high yielding or more adaptive cultivars. Interestingly, almost all key parents are today critically endangered varieties or have disappeared from our vineyards. All this data points to the importance and justification of preserving neglected, local varieties in germplasm repositories, in spite of their present low economic significance.

## Figures and Tables

**Table 1:** List of identified synonyms and duplicates at nine standard simple sequence repeat (SSR) loci.

No.	Accession 1	Accession 2	Accession 3	Accession 4	Matches
1	Plavina istarska (5IPT)	Surina (18IPT)			Maločrn, Plavina Maločrn [30]; Tükörszolo (P14#992), EuVDB *; Piccola nera [14]; Gnjet [37]
2	Garganja (6IPT)	Garganja (2-H)			Lipolist (B&H) [36]
3	Vela pergola (24IPT)	Popovo oko (20IPT)	Malvazija crvena (21IPT)	Unknown 5 (7IPT)	Prošip Bijela (B&H) [37]
4	Plavinica (8IPT)	Plavina (4-C)	Lun (3-K)		Plavka (B&H) [36]
5	Duranija (9IPT)	Draganela [57]			Duranja [30]
6	Jarbola (10IPT)	Jarbola (11-A)			
7	Borgonja (11IPT)				Frankovka (CZE041-24V0100221), EuVDB; Tamjanika crna [37]
8	Muškat ruža porečki (13IPT)				Rosenmuskateller (DEU098-2000-021), EuVDB; Cipro [30]
9	Hrvatica (14 IPT)	Kamenina (KRK02, KRK14)			Corredera (DEU098-1993-001), EuVDB
10	Teran (15IPT)	Teran (2-B)			Terrano [14]; Lambrusco picol ross [79]; Refošk [93]
11	Malvazija istarska (16IPT)	Malvazija istarska (9-D)	Malvazija istarska (12-A)		
12	Muškat momjanski (17IPT)				Muscat a petit grains (REF09); Muscat d’Alsace rose (FRA139-559Mtp8), EuVDB Moscatel Galego Branco ** [40]
13	Cimaroša (22IPT)				Velteliner rouge (FRA139-284Mtp4), EuVDB
14	Dolcin (23IPT)	Dolčin (2-D)			Vitouska (ITA368#SANOSVALDO/VC_10), EuVDB, [83,90] Vitovska [30]
15	Unknown 7 (25IPT)				Refosco peduncolo rosso (ITA362-1662), EuVDB
16	Pagadebit istarski (26IPT)	Kuč (3-C)	Maraština omiška (26-SK)		Mostosa = Empibotte bianco, [14]; Uva vacca [79]
17	Bašćan (KRK01)	Stara brajda (3-L)	Susac (10-E)	Crna Brajda (8-SK)	Bascina bijela (DEU098-1990-130), EuVDB
18	Plavčina (KRK04)	Plavčina (10-B)			
19	Ošljevina (KRK03)				Zelenika [29]; Rožica [30]
20	Troiščina (KRK08)	Trojiščina (11-B)			
21	Žumić (KRK)	Žumić (5-G)			
22	Brajdica (KRK)	Brajdica (2-J)			
23	Debejan (KRK)	Debejan (10-C)			
24	Gnjatonja(12-SK)	Dugovrst (5-B/2013)			
25	Svrdlovina (17-SK)	Svrdlovina (27-A)	Gustopupica bibinjska (4-i_novi )		
26	Okatica crna(19-SK)	Okatica vrgorska (VRG08)			
27	Palaruša bijela (22-SK)	Bumba bijela (8-K)	Medna bijela (internal acc.)		Žlozder [37]
28	Okatica bijela (23-SK)	Palaruša hvarska (4-D)	Palaruša viška (5-D)		
29	Silbijanac (25-SK)	Silbijanac (3-G)			
30	Divljak (ZAG01)	Ranfol (ZAG05)	Ranfol (ZAG27)	Ranfol (9-E)	
31	Dišeća ranina (ZAG08)	Dišeća ranina (9-K)			
32	Kavčina (ZAG10)				Kövidinka (P14#520), EuVDB
33	Belina Mala (ZAG11)				Biancghera [30]
34	Belina starohrvatska (ZAG16)				Gouais blanc (FRA139-211Mtp1), EuVDB; Heunisch weiss [88]; Branco Valente **[40]
35	Sokol (ZAG21)	Sokol (12-B)			Luglienga bianca (ITA360-501), EuVDB Caramela ** [40]
36	Volovina (ZAG26)				Moscato violetto [14]; Muscat rouge de Madère (ITA025-CO018), EuVDB
37	Modra kosovina (ZAG30)				Zimmettraube weiss (DEU098-1980-381), EuVDB
38	Moslavac (ZAG32)	Moslavac (9-L)			Furmint [14] Šipon [37]
39	Kozjak (ZAG42)				Coarna alba (DEU098-1991-188), EuVDB; Uva picciona [79]
40	Smudna belina (ZAG48)	Svjetljak (12-G)			
41	Beli Debejan (10-D)	Gegić (3-F)			
42	Verdić (11-F)				Teran bijeli [57]; Glera, Prosecco [29]; Beli Teran [30]; Prosecco tondo [89]
43	Petovka (3-E)	Cetinka (6-F)	Plavina bijela ninska (12-C)		
44	Malvasia dubrovačka (2-A)				Malvasia delle Lipari [94]
45	Drnekuša vela (3-D)	Glavanjuša (5-A)			
46	Cipar (3-J)				Grec rouge [14]; Rabigato Franco ** [40]
47	Maraština (4-A)				Malvasia del Chianti [14]; Pavlos [91]
48	Babić (4-B)	Babica plosnata (8-L)			
49	Lasina (4-E)	Vlaški crljenak Brač (5-J)			
50	Ninska crvena (4-K)	Šemperinka (8-G)	Vranac (7-G)	Vranac (15-F)	Vranac [14]
51	Pavicić (5-E)	Oskorušica (8-E)			
52	Bak (5-F)	Siložder (6-L)			
53	Bilan (5-H)				Racuk [29]
54	Beretinjok (5-I)	Topol (3-H_novi)			Bianco d’Alessano [11]
55	Muškat ruža omiški (5-K)				Muscat de Hambourg [14]
56	Muškat bijeli omiški (5-L)				Muscat d’Alexandrie [14]
57	Zlatarica vrgorska (6-C)				Kadarun IV, Surac IV [33]; Kadarun (B&H) [36]; Francavidda [11]
58	Pošip crni (6-D)	Šljiva (26-A)	Razaklija (28-B)		
59	Marinkovića grozje (6-I)				Dattier de Beyrouth = Afuz Ali [14]; Radovača [37]
60	Plavac mali (7-A)	Plavac mali sivi (7-C)			
61	Mijajuša (7-K)				Assouad karech [14]; Xerichi kokkino, EuVDB
62	Tribidrag (8-A)				Primitivo = Zinfandel [14] Kratošija [92]
63	Lelekuša (9-J)	Bratkovina crvena (30-A)			
64	Frmentum (6-H)				Santa Teresa [11]
65	Bratkovina bijela (29-A)	Pošipica (33-B/2013)	Stradunska [36]		Maruggio (Maresco)= Uva del Monaco [11]; Popetre [30]
66	Bljuzgavac (27-B_novi)				Blank blauer [14];
67	Lipovina (24-A/2013)				Harslevelu [14]
68	VRG10				Lisičina [60]
69	Kadarka				Olasz Kadarka [14]
70	Portugizac	Portugieser blau (REF12)			Portugais bleu [14]

* European *Vitis* Database [8], accessions numbers also presented when available. ** matches obtained through single nucleotide polymorphism (SNP) comparison.

**Table 2:** Genetic diversity parameters assessed for 36 SSR loci.

Locus	Allele Range (bp)	N	MD	N_a_	N_e_	N_ar_	H_e_	H_O_	F	HW	F null	PIC	P_(ID)unrelated_	P_(ID)sib_
VVS2	123–153	127	0	12	4.65	10.51	0.79	0.78	0.007	NS	0.0063	0.758	0.073	0.376
VVMD7	231–261	127	0	10	3.66	8.70	0.73	0.80	−0.095	NS	−0.0436	0.685	0.116	0.416
VVMD27	171–190	127	0	8	3.49	6.94	0.71	0.80	−0.115	NS	−0.0583	0.67	0.126	0.425
ssrVrZAG62	179–203	127	0	10	5.34	8.97	0.81	0.89	−0.095	NS	−0.0484	0.789	0.058	0.358
ssrVrZAG79	234–258	127	0	11	5.76	10.45	0.83	0.86	−0.039	NS	−0.0205	0.804	0.053	0.35
VVMD5	218–242	127	0	10	5.08	9.36	0.80	0.84	−0.049	NS	−0.0258	0.78	0.062	0.364
VVMD25	237–265	127	0	10	3.77	8.07	0.74	0.74	−0.007	NS	−0.0024	0.691	0.115	0.411
VVMD28	216–276	127	0	12	8.20	11.09	0.88	0.94	−0.067	ND	−0.0329	0.866	0.027	0.318
VVMD32	240–274	127	0	9	5.07	8.47	0.80	0.91	−0.128	NS	−0.0627	0.778	0.064	0.365
VViq52	101–111	126	0.79	5	2.78	4.71	0.64	0.71	−0.103	NS	−0.0544	0.57	0.2	0.48
VVIp31	192–216	127	0	12	7.18	11.18	0.86	0.95	−0.098	ND	−0.0476	0.845	0.035	0.328
VVip60	321–348	124	2.36	12	4.74	10.61	0.79	0.82	−0.032	NS	−0.0184	0.76	0.074	0.374
VMC1b11	185–215	125	1.57	10	4.88	9.62	0.80	0.85	−0.066	NS	−0.0362	0.774	0.063	0.368
VMC4f3	185–246	127	0	15	4.27	12.38	0.77	0.76	0.013	NS	0.0066	0.741	0.079	0.387
VVih54	166–205	126	0.79	12	4.11	10.15	0.76	0.81	−0.07	NS	−0.0341	0.732	0.084	0.393
VViv67	354–406	126	0.79	12	5.25	10.28	0.81	0.83	−0.03	NS	−0.0152	0.785	0.061	0.361
VVib01	307–325	127	0	5	2.79	5.00	0.64	0.67	−0.044	NS	−0.0205	0.58	0.19	0.477
VVMD24	224–235	127	0	6	2.55	5.92	0.61	0.61	0.003	NS	0.0046	0.559	0.202	0.496
VVMD21	244–281	126	0.79	7	2.60	5.92	0.62	0.71	−0.148	NS	−0.0712	0.57	0.193	0.491
VVIn16	167–177	127	0	5	2.45	4.98	0.59	0.58	0.03	NS	0.0116	0.523	0.235	0.513
VVIn73	272–285	127	0	7	1.47	5.14	0.32	0.32	0.011	ND	0.0095	0.287	0.496	0.715
VViv37	153–183	106	16.54	13	3.93	11.33	0.75	0.79	−0.063	NS	−0.0422	0.721	0.09	0.399
Vchr8b	115–169	125	1.57	15	7.41	13.59	0.87	0.69	0.205	**	0.1177	0.852	0.031	0.325
Vchr10b	145–154	126	0.79	3	2.45	3.00	0.59	0.66	−0.112	NS	−0.0525	0.505	0.254	0.517
Vchr14b	188–239	125	1.57	13	4.09	11.63	0.76	0.32	0.576	***	0.4073	0.728	0.087	0.394
Vchr4a	200–224	115	9.45	6	2.67	5.08	0.63	0.64	−0.03	NS	−0.0106	0.552	0.214	0.491
Vchr9a	108–142	126	0.79	8	4.26	7.36	0.77	0.83	−0.079	NS	−0.0355	0.73	0.09	0.39
Vchr16a	118–186	127	0	8	1.98	7.32	0.50	0.49	0.014	NS	0.012	0.457	0.293	0.576
Vchr19a	143–170	118	7.09	8	3.13	7.27	0.68	0.71	−0.047	NS	−0.0305	0.639	0.144	0.446
Vchr17a	197–205	119	6.3	2	1.83	2.00	0.45	0.38	0.168	NS	0.0914	0.351	0.401	0.623
Vchr18a	170–210	122	3.94	10	4.72	8.28	0.79	0.75	0.043	NS	0.0211	0.761	0.072	0.374
Vchr1b	112–128	122	3.94	4	2.85	3.97	0.65	0.61	0.065	NS	0.0277	0.578	0.194	0.474
Vchr7b	189–205	121	4.72	4	3.74	4.00	0.73	0.80	−0.094	NS	−0.0447	0.683	0.122	0.414
Vchr11b	169–181	109	14.17	6	4.05	5.38	0.75	0.78	−0.036	NS	−0.0188	0.708	0.106	0.4
Vchr12a	141–162	121	4.72	6	2.98	5.40	0.67	0.72	−0.082	NS	−0.0417	0.605	0.172	0.461
Vchr2b	130–139	62	51.18	4	1.64	4.00	0.39	0.37	0.047	ND	0.0171	0.348	0.414	0.659
Mean		122	3.718	9	3.9	7.72	0.70	0.71	−0.015		−0.0038	0.66		
Over loci													1.19 × 10^−34^	5.29 × 10^−14^

** significant at the 1 % level, *** significant at the 0.1 % level, NS = not significant, ND = not done. N = number of observed accessions; MD =% missing data; Na = number of alleles, Ne = effective number of alleles, Ar = allelic richness, He = expected heterozygosity, Ho = observed heterozygosity, F = fixation index, HW = exact test of departure from Hardy–Weinberg equilibrium, F null = estimated frequency of null alleles, PIC = polymorphic information content, P(ID) probability of identity = unrelated and sib .

**Table 3:** Genetic diversity parameters assessed for 45 SNP loci in the subset of 124 unique accessions of the Croatian grape germplasm.

SNP Locus *	N	MD	Na	N_e_	N_ar_	H_o_	H_e_	F	HW	MAF		PIC	P_(ID) unrelated_	P(_ID) sib_	F null
SNP1003_336	124	0	2	1.78	2.00	0.42	0.44	0.04	NS	A:	0.323	0.342	0.412	0.635	0.0207
SNP1015_67	124	0	2	1.46	2.00	0.35	0.32	−0.094	ND	C:	0.476	0.267	0.517	0.721	−0.0447
SNP1027_69	124	0	2	1.96	2.00	0.51	0.49	−0.036	NS	G:	0.266	0.37	0.38	0.6	−0.0175
SNP1035_226	124	0	2	1.39	2.00	0.31	0.28	−0.089	ND	T:	0.48	0.242	0.556	0.748	−0.0427
SNP1079_58	124	0	2	1.96	2.00	0.60	0.49	−0.219	NS	A:	0.246	0.37	0.38	0.6	−0.0988
SNP1119_176	124	0	2	1.98	2.00	0.48	0.49	0.02	NS	C:	0.375	0.372	0.378	0.598	0.01
SNP1127_70	124	0	2	1.64	2.00	0.44	0.39	−0.115	NS	A:	0.198	0.314	0.448	0.667	−0.0543
SNP1157_64	124	0	2	1.08	2.00	0.07	0.07	−0.038	ND	G:	0.266	0.068	0.867	0.932	−0.0097
SNP1215_138	124	0	2	2.00	2.00	0.53	0.50	−0.067	NS	C:	0.117	0.374	0.376	0.594	−0.0324
SNP1229_219	124	0	2	1.64	2.00	0.47	0.39	−0.197	NS	C:	0.016	0.314	0.448	0.667	−0.0899
SNP1323_155	124	0	2	1.69	2.00	0.48	0.41	−0.164	NS	G:	0.44	0.325	0.433	0.654	−0.0759
SNP1347_100	124	0	2	1.87	2.00	0.56	0.47	−0.198	NS	A:	0.351	0.357	0.395	0.616	−0.09
SNP1349_174	124	0	2	1.99	2.00	0.56	0.50	−0.116	NS	T:	0.431	0.374	0.376	0.595	−0.055
SNP1399_81	124	0	2	1.06	2.00	0.06	0.06	−0.029	ND	A:	0.286	0.053	0.895	0.946	−0.0062
SNP1411_565	124	0	2	1.32	2.00	0.27	0.24	−0.098	ND	C:	0.351	0.213	0.603	0.78	−0.0466
SNP1453_40	124	0	2	1.64	2.00	0.44	0.39	−0.115	NS	T:	0.379	0.314	0.448	0.667	−0.0543
SNP1471_179	124	0	2	1.26	2.00	0.23	0.21	−0.132	ND	C:	0.395	0.185	0.651	0.809	−0.0569
SNP1513_153	124	0	2	1.84	2.00	0.56	0.46	−0.222	NS	T:	0.302	0.352	0.4	0.622	−0.0998
SNP191_100	124	0	2	1.07	2.00	0.07	0.06	−0.033	ND	C:	0.169	0.06	0.881	0.939	−0.0079
SNP197_82	124	0	2	2.00	2.00	0.51	0.50	−0.016	NS	A:	0.367	0.375	0.375	0.594	−0.008
SNP227_191	124	0	2	1.85	2.00	0.48	0.46	−0.034	NS	T:	0.032	0.354	0.397	0.619	−0.0167
SNP259_199	123	0.81	2	1.76	2.00	0.46	0.43	−0.051	NS	G:	0.488	0.339	0.415	0.637	−0.025
SNP269_308	124	0	2	1.92	2.00	0.45	0.48	0.055	NS	C:	0.302	0.364	0.387	0.608	0.0284
SNP325_65	124	0	2	2.00	2.00	0.40	0.50	0.208	NS	A:	0.427	0.375	0.375	0.594	0.1163
SNP425_205	124	0	2	1.03	2.00	0.03	0.03	−0.016	ND	G:	0.472	0.031	0.938	0.969	−0.0023
SNP447_244	124	0	2	1.89	2.00	0.45	0.47	0.041	NS	A:	0.496	0.36	0.391	0.612	0.0207
SNP581_114	124	0	2	1.97	2.00	0.70	0.49	−0.424	***	A:	0.44	0.371	0.379	0.598	−0.1749
SNP593_149	124	0	2	1.84	2.00	0.51	0.46	−0.115	NS	T:	0.379	0.352	0.4	0.622	−0.0546
SNP613_315	124	0	2	1.34	2.00	0.27	0.25	−0.048	ND	A:	0.306	0.222	0.589	0.77	−0.0236
SNP697_296	124	0	2	1.30	2.00	0.25	0.23	−0.084	ND	A:	0.444	0.204	0.618	0.789	−0.0401
SNP819_210	124	0	2	1.59	2.00	0.44	0.37	−0.196	NS	G:	0.028	0.302	0.465	0.681	−0.0892
SNP829_281	124	0	2	1.97	2.00	0.57	0.49	−0.162	NS	C:	0.359	0.371	0.379	0.598	−0.075
SNP873_244	124	0	2	1.92	2.00	0.50	0.48	−0.046	NS	C:	0.351	0.364	0.387	0.608	−0.0225
SNP879_308	124	0	2	2.00	2.00	0.57	0.50	−0.146	NS	A:	0.488	0.375	0.375	0.594	−0.068
SNP895_382	124	0	2	1.89	2.00	0.52	0.47	−0.096	NS	A:	0.113	0.36	0.391	0.612	−0.046
SNP945_88	124	0	2	2.00	2.00	0.52	0.50	−0.049	NS	T:	0.266	0.375	0.375	0.594	−0.0239
SNP947_288	124	0	2	1.99	2.00	0.63	0.50	−0.263	NS	A:	0.141	0.374	0.376	0.595	−0.1163
VVI_10113	124	0	2	1.89	2.00	0.40	0.47	0.143	NS	A:	0.317	0.36	0.391	0.612	0.0772
VVI_10329	124	0	2	1.88	2.00	0.27	0.47	0.432	***	T:	0.149	0.359	0.392	0.614	0.2757
VVI_10353	124	0	2	1.84	2.00	0.49	0.46	−0.08	NS	A:	0.468	0.352	0.4	0.622	−0.0385
VVI_10992	124	0	2	1.73	2.00	0.41	0.42	0.025	NS	G:	0.387	0.333	0.423	0.645	0.0128
VVI_12882	124	0	2	1.73	2.00	0.35	0.42	0.178	NS	A:	0.036	0.333	0.423	0.645	0.0978
VVI_1617	124	0	2	1.74	2.00	0.52	0.43	−0.214	NS	G:	0.395	0.335	0.421	0.643	−0.0967
VVI_9227	124	0	2	1.25	2.00	0.19	0.20	0.034	ND	G:	0.133	0.18	0.66	0.815	0.0172
VVI_9920	124	0	2	1.90	2.00	0.48	0.48	−0.02	NS	G:	0.379	0.362	0.389	0.61	−0.0098

* SNP579_187e is not shown due to being non polymorphic. *** significant at the 0.1 % level; N = number of observed accessions; MD = % missing data; Na = number of alleles, Ne = effective number of alleles, Nar = allelic richness, Ho = observed heterozygosity, He = expected heterozygosity, F = fixation index, HW = exact test of departure from Hardy–Weinberg equilibrium, MAF = minor allele frequency, PIC = polymorphic information content, P(ID) probability of identity = unrelated and sib; F null = estimated frequency of null alleles.

**Table 4:** Comparison of genetic diversity statistics assessed for SSR and SNP sets recommended for routine fingerprinting.

Marker Type	N loci	N	MD	A	N_a_	H_o_	H_e_	PIC	cum P_(ID)unrelated_	cum P_(ID)sib_
SSR	9 *	127	0	92	10.22	0.84	0.79	0.76	4.07 × 10^−11^	1.44 × 10^−04^
SNP	45	124	0.02%	90	2	0.416	0.39	0.31	4.57 × 10^−16^	1.00 × 10^−08^

* set of nine recommended SSR loci [7]. N = number of observed accessions, MD = missing data, A = total number of amplified alleles, Na = average number of alleles per locus, Ho = observed heterozygosity, He = expected heterozygosity, PIC = polymorphic information content, P(ID) probability of identity = unrelated and sib.

**Table 5:** Proposed full parentages of Croatian germplasm based on 20 SSR loci.

Offspring	First Candidate Parent	Second Candidate Parent	SSR Total/ Mismatch	LOD	add. SSR Total/ Mismatch	SNP	Previously Reported
Sokol (n.d.) *	Bermestia bianca	Madeleine Salomon = Agostenga blanc	20/0	46.01			[14]
Dolcin (D)	Verdić (D)	Maraština (D)	20/0	40.19	14/0	45/45	[19,90]
Ljutun (D)	Plavac mali (D)	Bombino bianco (D)	20/0	40.04	14/0		
Krstičevica (n.d.)	Plavina (C)	Bombino bianco (D)	20/0	39.62	13/0		
Plavina (C)	Tribidrag (D)	Verdeca = Lagorthi	20/0	37.72			[12,14]
Kurtelaška (D)	Kuč (A)	♀ Bombino bianco (D)	20/0	37.56	14/0		[14]
Belina svetokriška (C)	Alba imputotato	Belina starohrvatska (C)	19/0	36.38			
Debit (D)	Lasina (C)	♀ Bombino bianco (D)	20/0	36.34	8/0		[14]
Kozjak b. (C)	Bulanyi	Csomorika	18/0	36.19			
Ninčuša (D)	Plavac mali (D)	Bombino bianco (D)	20/0	35.70	14/0		[14]
Beli Debejan (D)	Privlačka bilina (n.d.)	♀ Bombino bianco (D)	19/0	35.35	15/0		
Muškatel (n.d.)	Dattier de Beyrouth = Afuz Ali	Perle de Csaba )	20/0	34.97			
Mejsko bijelo (A)	Duranija (A)	Žumić (A)	20/0	34.77	8/1	45/1	
Lipovina (n.d.)	Moslavac (C)	Tzimliansky belyi	20/1	34.67			
Pošip b. (C)	♀ Zlatarica blatska (C)	Bratkovina bijela (D)	19/0	34.47	13/1	45/1	[63]
Moslavac (C)	Alba imputotato	Belina starohrvatska (C)	20/0	34.43			
Glavinuša (A)	Plavac mali (D)	♀ Vugava (A)	20/0	34.05	14/2		[14]
Crnka (D)	Plavac mali (D)	Bratkovina crvena (D)	20/0	33.30	14/0	45/0	
Volovina (n.d.)	Sciaccarello = Mammolo	Muškat momjanski=Muscat a pet. grains	20/0	32.94			[14]
Belina šemnička (C)	Kovacs Kreger	Belina starohrvatska (C)	18/0	32.51			
Bljuzgavac (D)	Bratkovina crvena (D)	Gyöngy feher	20/0	32.31			[14]
Plavec žuti (C)	Bljuzgavac (D)	♀ Belina starohrvatska (C)	20/0	31.63	12/1		
Plavina istarska (C)	Pinella bianca	Bljuzgavac (D)	20/0	29.55			
Ranfol (C)	Bljuzgavac (D)	♀ Belina starohrvatska (C)	20/0	28.84	13/2	45/0	[14]
Svjetljak (C)	Bljuzgavac (D)	♀ Belina starohrvatska (C)	20/0	27.80	12/2	45/1	

* chlorotype; n.d. = not determined.

**Table 6:** Examples of possible direct (first-degree) relationships identified within Croatian grape germplasm using 20 SSR.

Cultivar 1	Cultivar 2	SSR Genotyped/ Mismatch	LOD Score	Comments
Bljuzgavac	Žlahtina	20/0	13.22	Possible only if cultivar 1 is genitor of cultivar 2
Bašćan	Bombino bianco	20/0	12.31	
Belina Hižakovec	Kozjak bijeli	19/0	15.42	
Belina Mala	Moslavac	20/0	7.63	
Belina Mala	Sacy [14]	20/0	9.16	
Belina Šemnička	Kozjak bijeli	18/0	12.13	Cultivar 2 grandparent of cultivar 1
Bogdanuša	Palagružanka	20/0	11.85	
Bombino bianco	Glavanjuša	20/0	10.68	
Bombino bianco	Mladenka	20/0	15.63	
Bombino bianco	Žutozelen	19/1	13.61	
Bratkovina bijela	Pošip crni	19/0	13.61	
Dišeća ranina	Kozjak bijeli	19/0	7.24	Possible only if cultivar 2 is genitor of cultivar 1
Dišeća ranina	Urmi dinka [14]	19/0	10.8	
Draganela	Plavčina	20/0	15.22	
Drnekuša mala	Plavac mali	20/0	15.66	
Glavanjuša	Plavac mali	20/0	20.41	
Jarbola	Belina starohrvatska	20/0	9.37	
Klešćec	Argant [14]	20/0	19.87	
Malvazija župska	Belina starohrvatska	20/0	11.45	
Plava lovora	Plavac mali	19/0	16.37	
Plavac mali	Siložder	20/0	15.24	
Plavac mali	Šemperinka bijela	18/0	16.1	
Plavac mali	Vugava b. omiška	19/0	17.87	
Plavac mali	Zelenjak	20/0	18.64	
Pošip crni	Vugava bijela omiška	19/0	11.38	
Pršljivka	Alba imputotato [14]	20/0	11.28	
Sansigot	Tribidrag	20/0	13.87	
Silbijanac	Alba imputotato [14]	20/0	17.97	
Škrlet	Argant [14]	20/0	18.17	
Teran	Barbera [14]	17/0	6.05	
Teran	Greco nero [14]	20/0	19.77	

## Supplementary Materials

The following are available online at https://www.mdpi.com/2073-4425/11/7/737/s1, Figure S1: Determination of K values according to Reference [109], Evanno et al. (2005). The rate of change of the posterior probability of the data given the number of subgroups is plotted against K. The first peak (K = 5) corresponds to the optimum number of subgroups. Table S1: List of 212 Vitis vinifera L. subsp. sativa accessions including their codes, presumed varietal names, skin color, grape utilization, sampling collection, main cultivation region, conservation status and types of analyses conducted, Table S2: List of reference Vitis accessions, Table S3: Primer sequences, chromosome positions, fluorophore, range, associated annealing temperatures and PCR and capillary electrophoresis arrangement of SSR and cpSSR loci used in this study, Table S4: 47 SNP loci recommended for identification analyzed in this study and expected polymorphism, Table S5: Determined status of analyzed accessions, Table S6: Microsatellite profiles for 36 SSR loci and chlorotypes for 127 presumed Croatian varieties, Table S7: SSR allele frequencies for 127 presumed Croatian varieties, Table S8: 46 SNP profiles for 124 presumed Croatian varieties, Table S9: Determined chlorotype haplotypes based on 4 cpSSR markers. Table S10: Genome share of all varieties from the putative Croatian set in each of the five proposed gene pools. Q values > 0.75 are highlighted, Table S11: AMOVA results and pairwise population ΦPT values, Table S12: Complete list of Croatian cultivars and other Vitis vinifera cultivars in possible direct (first-degree) relationship.
