# Supplementary material for: Genetic Diversity, Population Structure, and Parentage Analysis of Croatian Grapevine Germplasm
Source: Genes (Basel). 2020 Jul 2;11(7):737. doi: 10.3390/genes11070737 (PMC7397172; doi:10.3390/genes11070737)
Supplement: Supplementary file 1 [file genes-11-00737-s001.zip › Figure S1.docx]

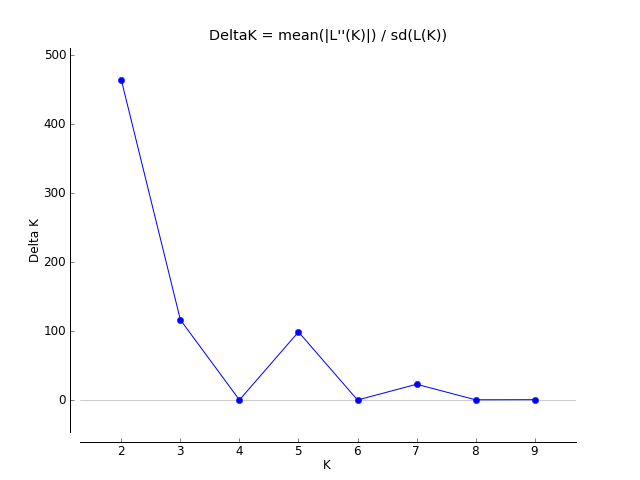


**Figure S1.** Determination of K values according to [109] Evanno et al. (2005). The rate of change of the posterior probability of the data given the number of subgroups is plotted against K. The first peak (K = 5) corresponds to the optimum number of subgroups.
